# The Diagnostic Significance of Convulsions

**Published:** 1913-10

**Authors:** Lewis M. Gaines

**Affiliations:** Prof. Neurology and Mental Diseases, Atlanta Medical College, Atlanta, Ga.


### THE DIAGNOSTIC SIGNIFICANCE OF CONVULSIONS.

By Lewis M. Gaines, M. D., Prof. Neurology and Mental Diseases, Atlanta Medical College, Atlanta, Ga.

Few symptoms are more striking to the medical observer, or more terrifying to the layman than convulsions. The hapless victim appears to be in the grasp of some hideous and ruthless force that threatens his very being with destruction. Small wonder it was that in ancient times persons with convulsions were supposed to be possessed of demons.

In this discussion, I am dealing only with general convulsions, usually accompanied by loss of consciousness. In such cases certain features are present regardless of the cause of the convulsions. These may be briefly summarized. If the patient is not recumbent, he falls sometimes with injury to himself. In volvement of the muscles of the face and jaws, gives rise to horrible grimaces, excessive saliva secretion which is churned into a froth, streaked with blood if the tongue or cheeks are caught between the clashing jaws. If the respiratory muscles are involved there is more or less cyanosis. The movements are clonic in character, limited in range and without purpose. Rigidity is present. In the epileptic seizure the clonus succeeds a brief tetanic stage. The sphincters often relax, with escape of urine and sometimes feces. The reflexes are usually lost during and for some hours after the convulsion. However, the Babinski is frequently an exception, and is probably without significance. After the return of consciousness, the patients are usually dull and stupid, often somnolent. Occasionally, particularly in epilepsy, they are excited or maniacal. One of my cases ran five miles at top speed, pursued by frenzied relatives after an epileptic fit. During such sequelae the patients are not responsible for their actions. Epileptics have thus been known to commit atrocious crimes, of which they had no knowledge after recovery from the attack. In regard to duration of convulsions, they usually last only a few minutes, but if unchecked may continue for hours, or one convulsion may succeed another with very brief intermission until the patient succumbs.

Such a seizure causes a rise of temperature of several degrees. It is important to remember that albumin is very frequently present in the urine after a convulsion, no matter what the cause. The amount may be large and accompanied by tube casts. Such findings do not permit a diagnosis of uremia, and taken alone are of no value.

The physician when he encounters a case with convulsions should ask himself two questions: First, under what conditions do convulsions occur; second, what data should be obtained necessary to determine which condition is responsible for the case under observation. It is well to keep in mind a classification scheme of the causes of convulsions, not only as an aid to memory, but because it is of some help in diagnosis. The following division is, I think, valuable:

1. Convulsions occurring in infants and children, due to the following causes: Hereditary Syphilis, Congenital Heart Disease, Cerebral Palsy, Onset of Acute Fevers, Digestive Disturbances, Meningitis, Drug Poisoning, Enlarged Thymus, Idiocy, Rickets, Epilepsy, and cerebral congenital anomalies.

Convulsions occurring in youth and adult life, due to following causes: Epilepsy, Jacksonian Epilepsy, Uraemia, Eclampsia, certain forms of severe heart disease, Asphyxia, Stokes-Adams Disease, occasionally in acute infections, as Malaria, Meningitis, certain cerebral lesions for example, Apoplexy and Intra-cranial Growth, Meningeal Hemorrhage, General Paresis, Cerebral Syphilis, Chronic Alcoholism, occasionally in cases of Acute Mania, Hysteria, and, it should not be forgotten in Malingering.

3. Unilateral convulsions may occur in Apoplexy, Intracranial Growth, Meningitis, Epilepsy, Jacksonian Epilepsy, and Disseminated Sclerosis.

Few features of a paper on a medical or other subject are more wearisome than long systems of classification, and I by no means propose to discuss separately the differential diagnosis of the various diseases just enumerated, in which convulsions may occur. I have, however, ventured to read the list in order to show what a large number of affections, very diverse in character, may be responsible for the occurrence of convulsions. I do not think I have exhausted the list, even yet, for there are probably other poisons of various kinds which may act on the central nervous system in such a way as to produce a convulsion.

I now come to the second part of my paper. What data should be obtained which will be useful in determining the condition responsible for a given case of convulsions. It is of course evident that the occurrence of convulsions is in itself of not very marked diagnostic value. It forms only one of a group of symptoms and physical signs necessary to a diagnosis.

In the first place the form of the convulsion must be studied. Is it general or unilateral? Does it present the sequence of tonic and clonic stages such as is seen in epilepsy typically and for this reason known as "epileptiform"? If so, the probabilities are narrowed. Is the convulsion hysteroid in character, and as such noisy, protracted and often with purposive movements, and evident incomplete suppression of consciousness? Finally, is the convulsion of the type assumed by malingerers? These misguided persons attempt a stimulation of the epileptic fit, but close observation will reveal that the face is red rather than cyanosed, that consciousness is not lost, that frequently painful impressions properly administered will cause flinching, that the sphincters are not relaxed. Occasionally a malingerer will display wonderful fortitude in his attempts to successfully carry through his deception. I have seen such an individual endure without flinching strong supra-orbital pressure and even touching of the cornea, but quickly change his attitude to one of belligerency when the administration of ether was begun. This individual then freely admitted that his only reason for simulation was his desire to have a warm bed in the hospital on what was a very cold winter night.

Having observed the form of convulsion, the next step is the obtaining of as complete a history, past and present, as posable. Aside from the physician's own observation of the age and sex of the patient, the particular points of inquiry should be concerning the habits and temperament of the patient, the existence of previous attacks, if a woman, whether childbirth was recent or is imminent, if a child, a history if digestive disturbances, and finally a careful questioning of relatives or friends where such are available, concerning any previous departure from a condition of health. The temperature should be obtained per rectum. This is of comparatively little aid, but there is less apt to be fever in epilepsy, hysteria, alcoholism for example than in meningitis or eclampsia. Likewise the urinalysis is of no very great diagnostic significance, but we would hardly expect to find albumin and casts in such a condition as hysteria, unless the patient happened to have a coexistent kidney lesion. The blood should always be examined with reference to leucocytosis, which we would expect to find in meningitis, uraemia, eclampsia, and expect to be absent in hysteria, alcoholism and many other conditions. One should also examine the stained specimen for the stippling of the reds so often found in lead poisoning, and for the presence of malarial parasites, and other anomalies. Arterial hypertension should be determined after the subsidence of the seizure, as we should expect to find this condition in eclampsia, apoplexy, uraemia, though too much reliance should not be placed in its existence as a diagnostic factor. Paralysis should be searched for some time after the subsidence of the convulsion, for its occurrence immediately and for several hours after the attack is of little help. It is important to remember that such causes as eclampsia, uraemia, meningitis and hysteria are efficient in bringing about more or less transient paralysis. The hysterical paralyses are of special interest in that they may exist for days, months or years, often accompanied by areas of anaestheia irregularly situated, and by contraction of the visual field, especially with reference to the perception of colors. In addition, various accidents and stigmata of hysteria are of importance in determining its diagnosis. Very important information as to the cause of convulsions is frequently to be obtained from examination of the fundus oculi with the ophthalmoscope. Thus retinitis is frequently in uraemia, and choked disc in intra-cranial growths. A negative finding, however, does not rule out these conditions. Finally, of the greatest importance in every doubtful case of convulsions is the information to be gained by careful study of the spinal fluid obtained by lumbar puncture. The fluid should be examined for micro-organisms, which have given rise to one of the forms of meningitis. A Wassermann should be done and the number of cells counted, the globulin tested for and Fehling's test made. The results give us definite information concerning the presence or absence of syphilis of the nervous system. It should be remembered in this connection that general paresis is now to be considered syphilis of the brain, and that it is not unusual to see epileptiform convulsions at various stages of the disease. Other clinical symptoms are to be remembered, but they may be very slight, and easily overlooked, even at the time the convulsion appears. Symptoms are probably dependent upon the anatomical area in the cortex in which the spirochaetae are proliferating. Should this occur in the motor area largely, convulsions may occur as an early symptom.

It is hardly necessary to say that a careful physical examination should be made. Such conditions as pneumonia, meningitis, various forms of heart lesions, pregnancy for example, could thus be determined to be present or absent. Very often a careful physical examination, omitting no part of the body, will be more effective in establishing a diagnosis, than refined laboratory and special tests. (Thus, recently I was called to see a young man who had dismissed his physician whom he had first called, because he was obtaining no relief for a continued fever. He was being treated for malaria. The patient proved to have a perineal abscess, which I immediately turned over to the tender mercies of a surgeon. Evacuation produced a cure in a very short time.) In a case of convulsions, a thorough physical examination may save an inexcusable mistake in diagnosis.

It should be remembered, however, that in not a few cases of convulsions, it is impossible to make a positive diagnosis immediately. Such a case may have to be studied at length, the various data I have spoken of obtained, the evidence weighed, and the result of treatment watched before a definite cause can be assigned.

I think we might be justified in these conclusions:

1. Convulsions occur as a symptom at the onset or during the course of a large number of diseases of very diverse etiology.

2. A true convulsion is an indication of a powerful toxic or perhaps occasionally mechanical effect on the cerebral cortex, particularly the motor area, and constitutes a challenge to the physician to determine the nature of the irritant.

3. Since convulsions occur as a symptom in so many conditions, their diagnostic significance is slight, unless other information is obtained. Such information is to be gleaned by observing the form of the convulsion, careful complete physical examination (perhaps oft repeated), a complete history, past and present, and study of the blood, urine, spinal fluid, determination of persistent paralysis, examination of the fundus oculi, and by observation of the results of treatment.

619-620 Empire Bldg.

